# Supplementary material for: Evaporative water loss of 1.42 million global lakes
Source: Nat Commun. 2022 Jun 28;13:3686. doi: 10.1038/s41467-022-31125-6 (PMC9240014; doi:10.1038/s41467-022-31125-6)
Supplement: Supplementary file 1 — Supplementary Information [file 41467_2022_31125_MOESM1_ESM.pdf]

Supporting information for

Evaporative water loss of 1.42 million global lakes

Gang Zhao[1,2], Yao Li[2], Liming Zhou[3], Huilin Gao[2*]

[1]Department of Global Ecology, Carnegie Institution for Science, Stanford, CA 94305, USA

[2]Zachry Department of Civil and Environmental Engineering, Texas A&M University, College Station, TX 77843, USA

[3]Department of Atmospheric and Environmental Sciences, State University of New York at Albany, Albany, NY 12222, USA

[*]Corresponding author:

Huilin Gao

Associate Professor

Zachry Department of Civil and Environmental Engineering

Texas A&M University

College Station, TX 77843-3136

Email: hgao@civil.tamu.edu

Supplementary online material

Fig. 1 to 15

Note 1

Table 1 to 3

References

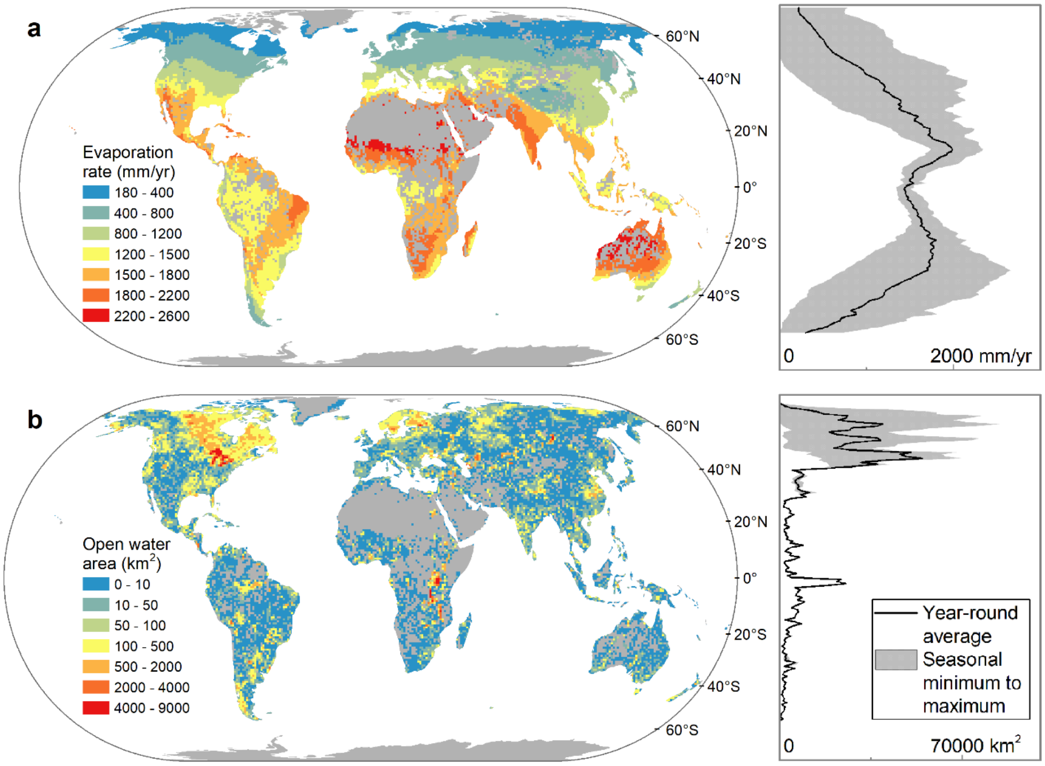

Supplementary Fig. 1.
Global average (a) evaporation rate (E_{lake}), (b) open water area (A_o) from 1985 to 2018, and their latitudinal distribution. The original results for 1.42 million lakes and reservoirs were aggregated to equal-area grids under the World Eckert IV projection for better illustration. The grey color in the maps indicates no data. The global heterogeneity of the evaporation rate is a result of the spatial patterns of the four governing variables (i.e., shortwave radiation, air temperature, humidity, and wind speed), and is consistent with global potential evapotranspiration maps (Supplementary Fig. 8).

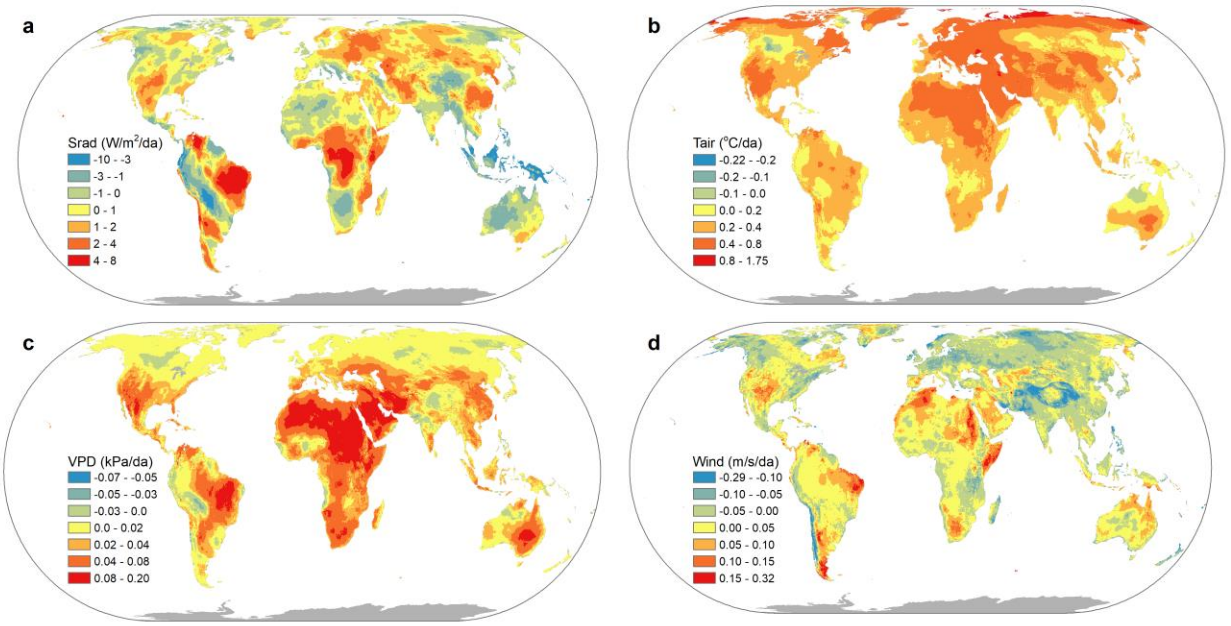

Supplementary Fig. 2.

Long-term trend for a) downward surface shortwave radiation, b) surface air temperature, c) surface vapor pressure deficit, and d) 10m wind speed. These values were averaged for the three meteorological datasets: TerraClimate, GLDAS, and ERA5.

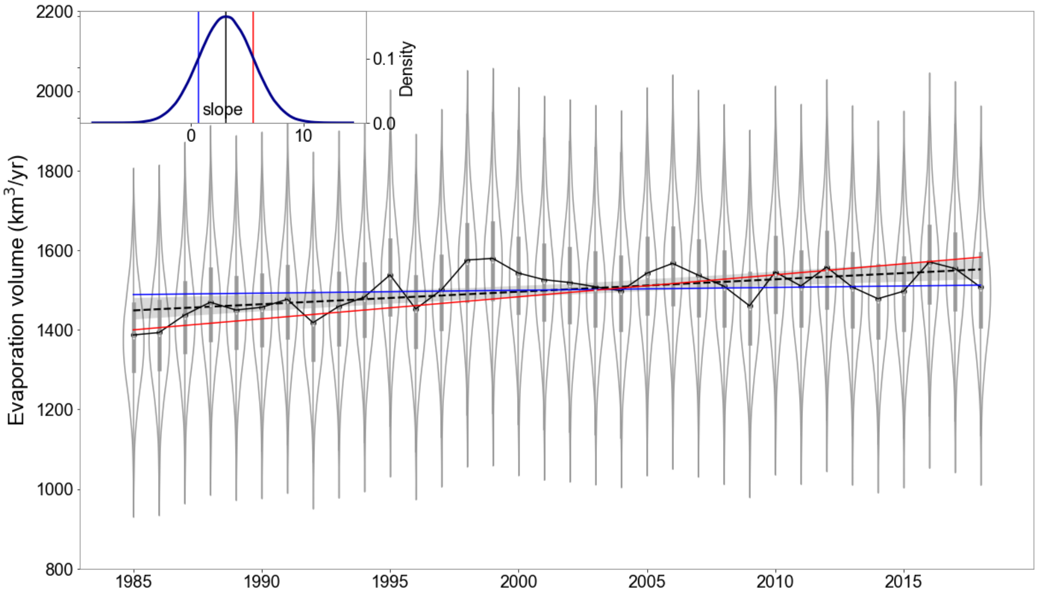

Supplementary Fig. 3.

The trend uncertainty of the evaporation volume for the 1.42 million lakes. The Monte Carlo simulation was employed by randomly sampling from the Gaussian distribution of evaporation volume for each year with the prescribed standard deviation of 9.93%. After 1 million cycles of Monte Carlo run, the slope uncertainty has been quantified as ±2.4 km^3 $year^{-1}$.

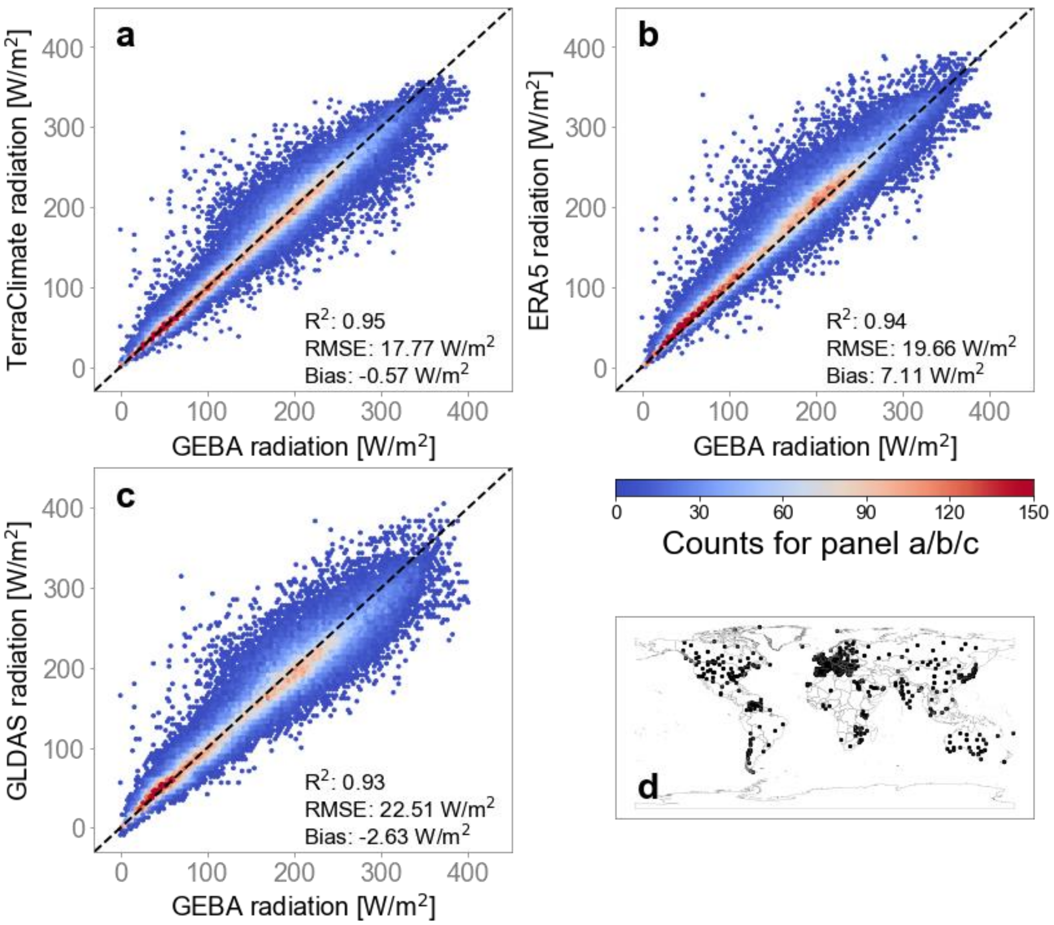

Supplementary Fig. 4.
Validation of surface downward shortwave radiation from three reanalysis datasets including a) TerraClimate, b) ERA5, and c) GLDAS using d) 657 ground stations from Global Energy Balance Archive (GEBA).

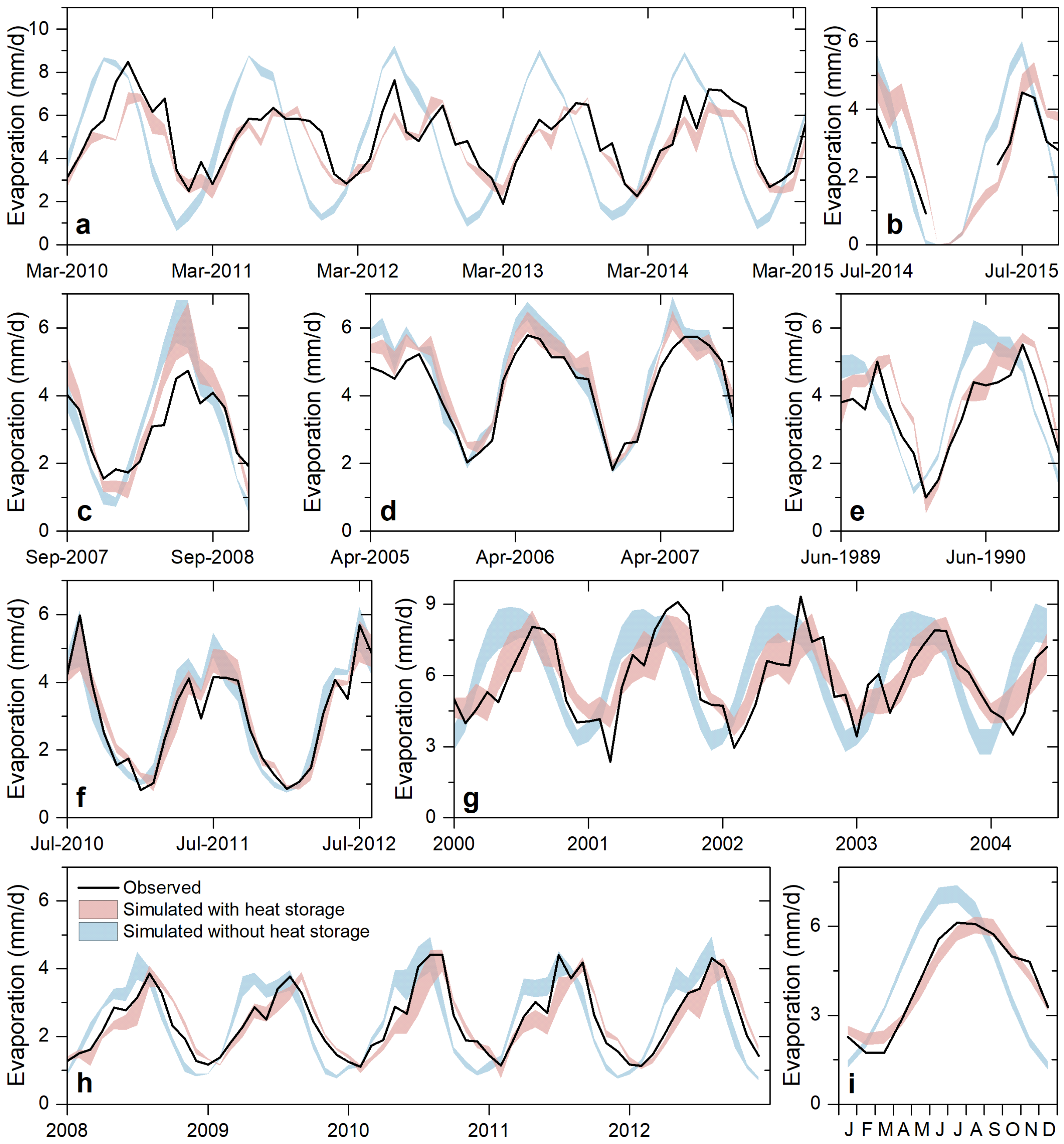

Supplementary Fig. 5.

Evaporation rate validation for a) Lake Mead, USA, b) White Bear Lake, USA, c) Ross Barnett Reservoir, USA, d) Lake Calm, USA, e) Lake Five-O, USA, f) Lake Taihu, China, g) Lake Nasser, Egypt, h) Lake Kasumigaura, Japan, and i) Lake Kinneret, Israel.

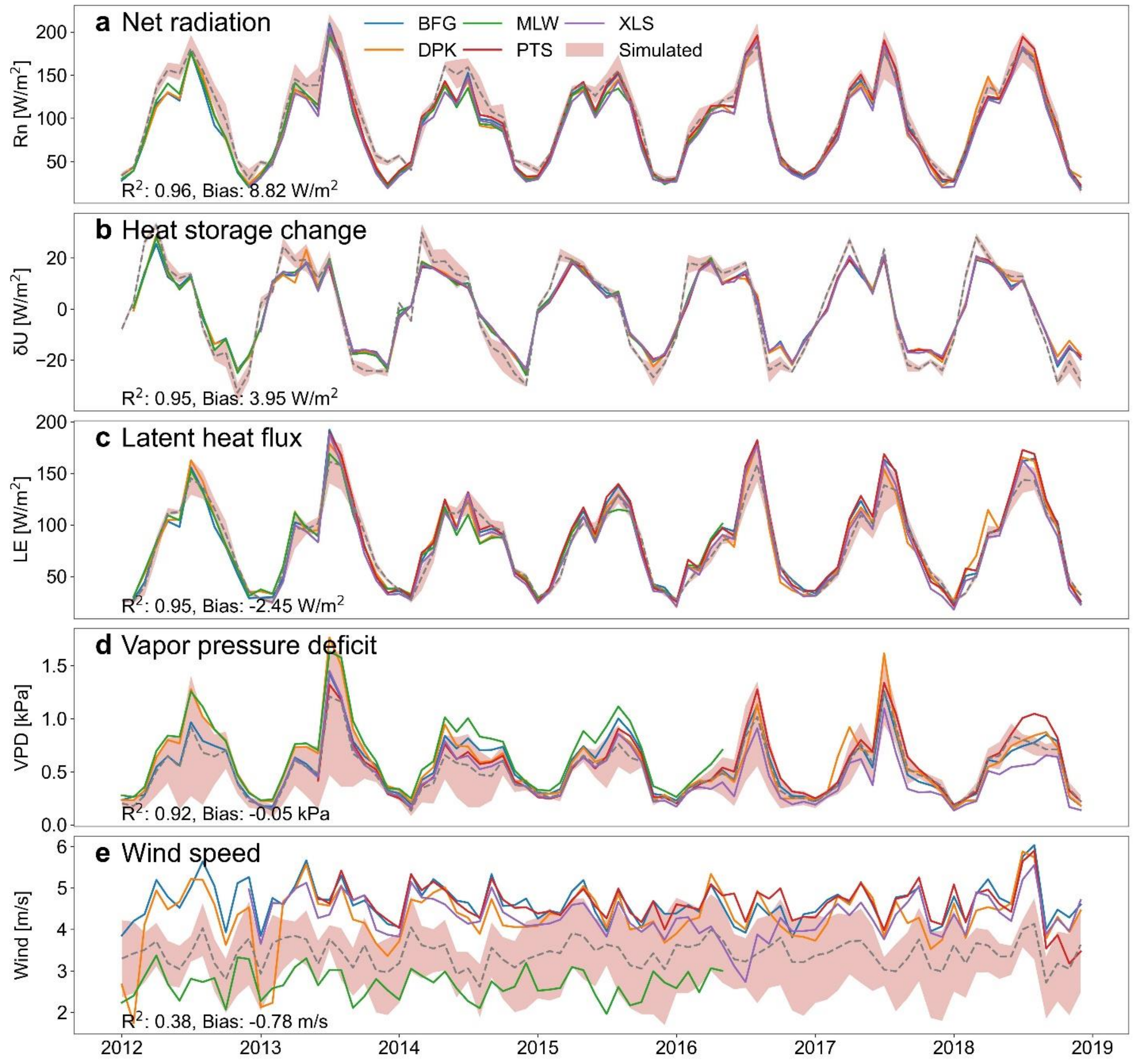

Supplementary Fig. 6.

Evaluation of a) net radiation, b) heat storage change, c) latent heat flux, d) vapor pressure deficit, and e) wind speed at Lake Taihu (average depth of 1.9m) at a monthly scale. The observed data were collected from Zhang et al. (2020). The shaded area represents the estimation uncertainty from different input forcing datasets (i.e., TerraClimate, ERA5, and GLDAS) and the dashed line represents the average values. The wind speed from the reanalysis datasets have been converted from the 10m reanalysis height to the heights of the in-situ data measurements according to the wind profile power law.

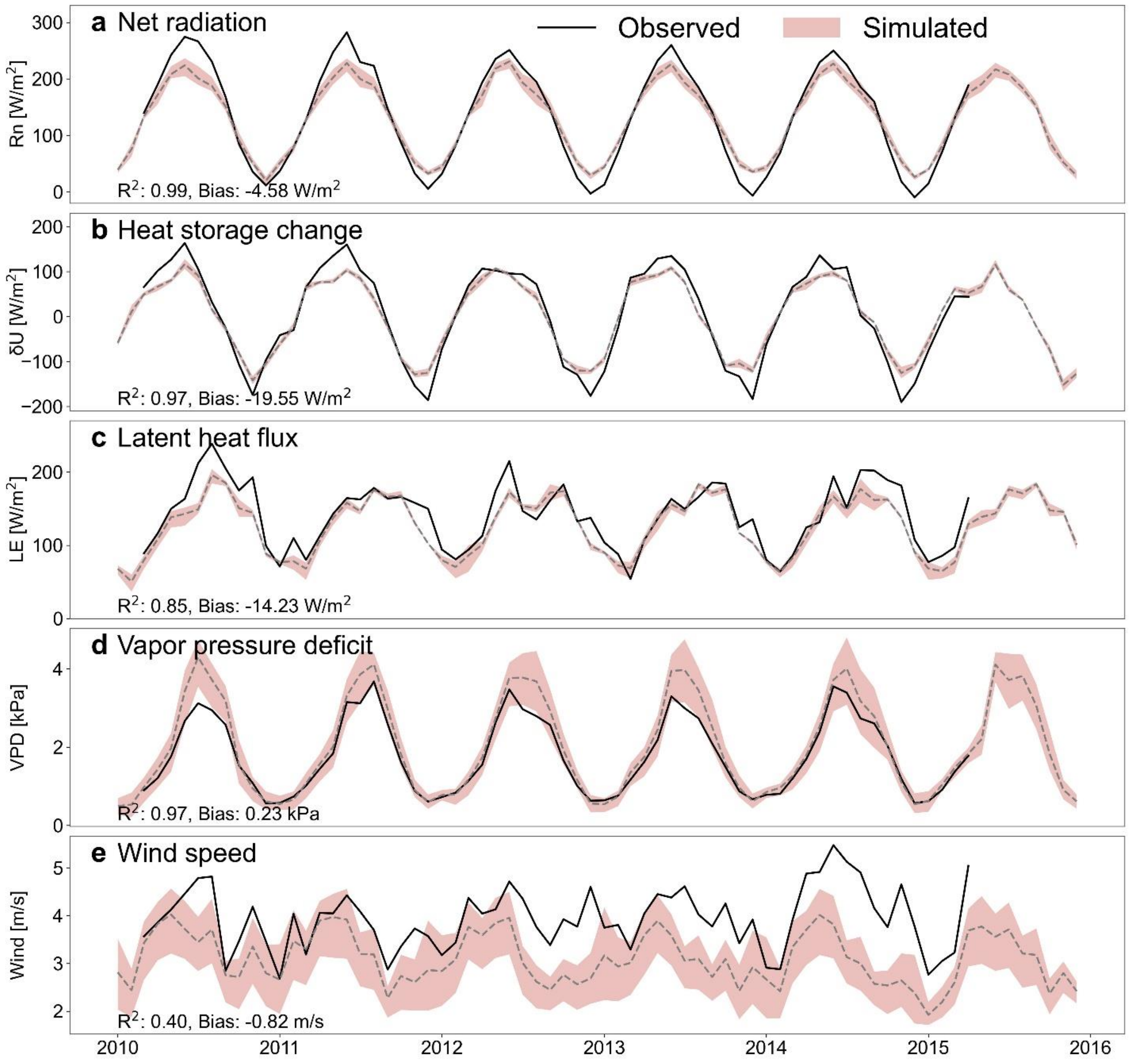

Supplementary Fig. 7.

Evaluation of a) net radiation, b) heat storage change, c) latent heat flux, d) vapor pressure deficit, and e) wind speed at Lake Mead (average depth >50m) at a monthly scale. The observed data were collected from Moreo (2015). The shaded area represents the estimation uncertainty from different input forcing datasets (i.e., TerraClimate, ERA5, and GLDAS) and the dashed line represents the average values. The wind speed from the reanalysis datasets have been converted from the 10m reanalysis height to the heights of in-situ data measurements according to the wind profile power law.

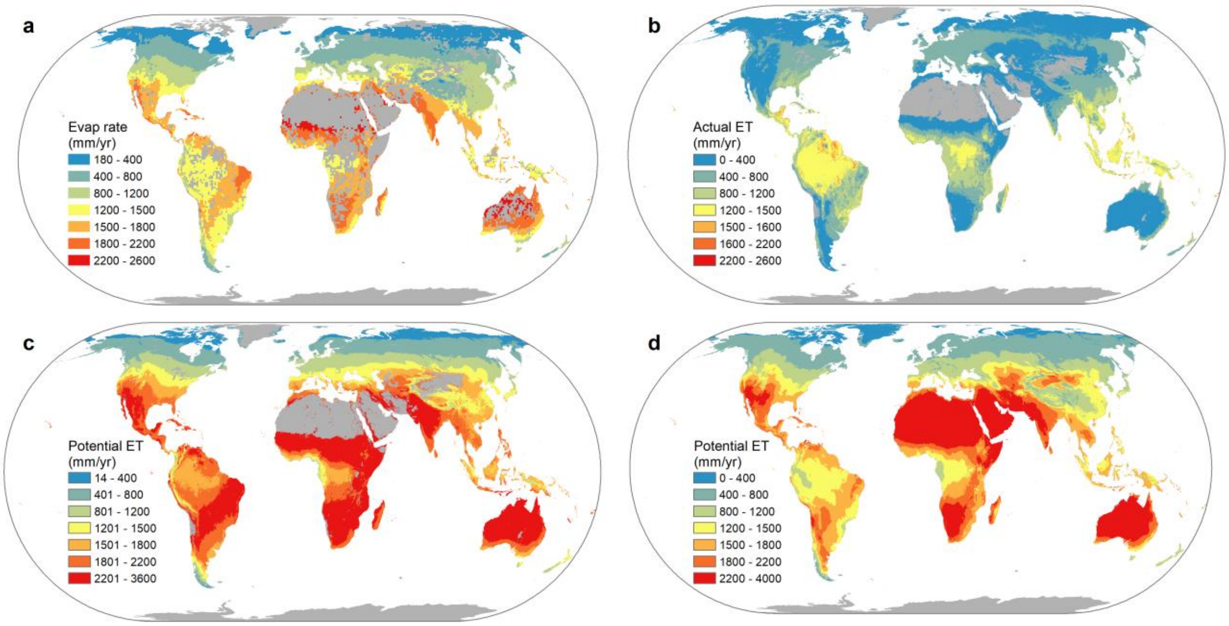

Supplementary Fig. 8.
Long-term average evaporation rate for a) open water (this study), b) actual evapotranspiration (from MOD16A2), c) potential evapotranspiration (from MOD16A2), and d) potential evapotranspiration (Trabucco and Zomer, 2019).

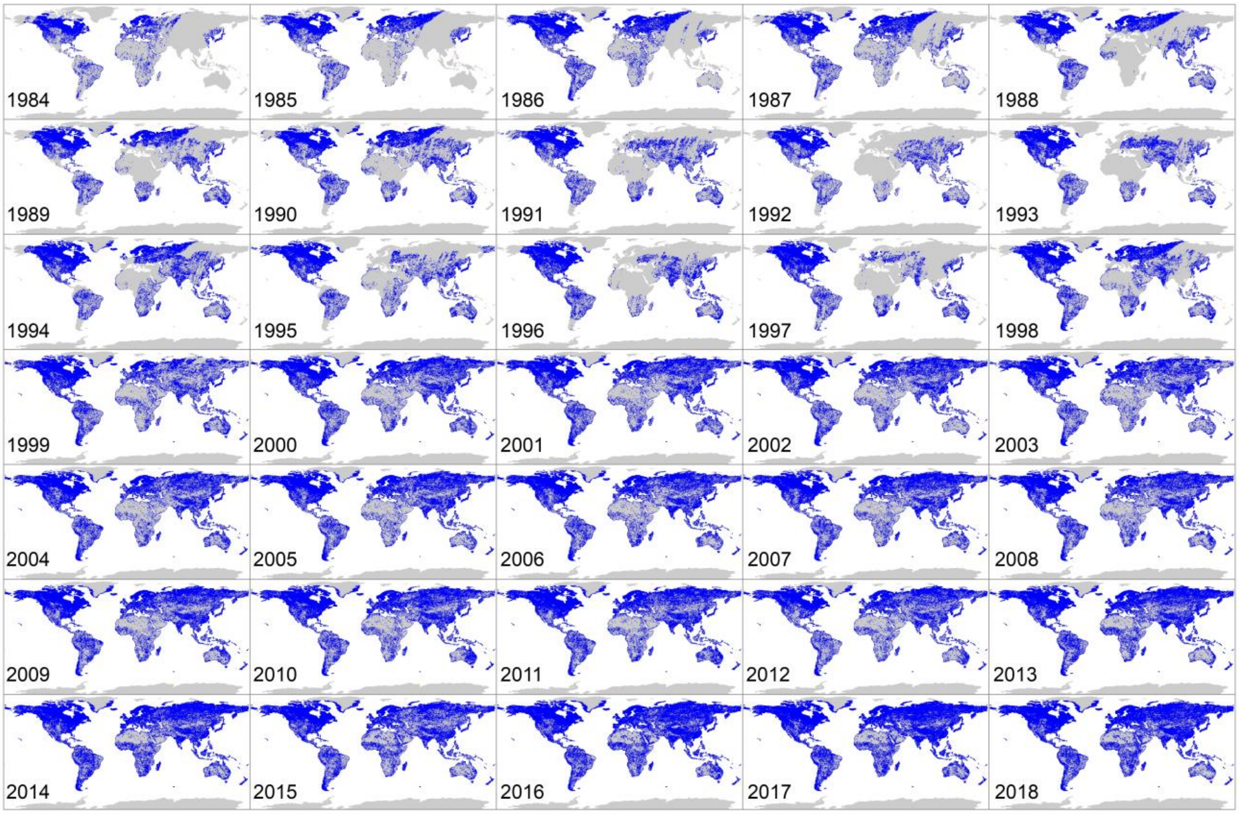

Supplementary Fig. 9.
Coverage of global surface water dataset (GSWD) annual water maps from 1984 to 2018. Some regions have shown limited coverage before 1999, which is the launch year of Landsat 7.

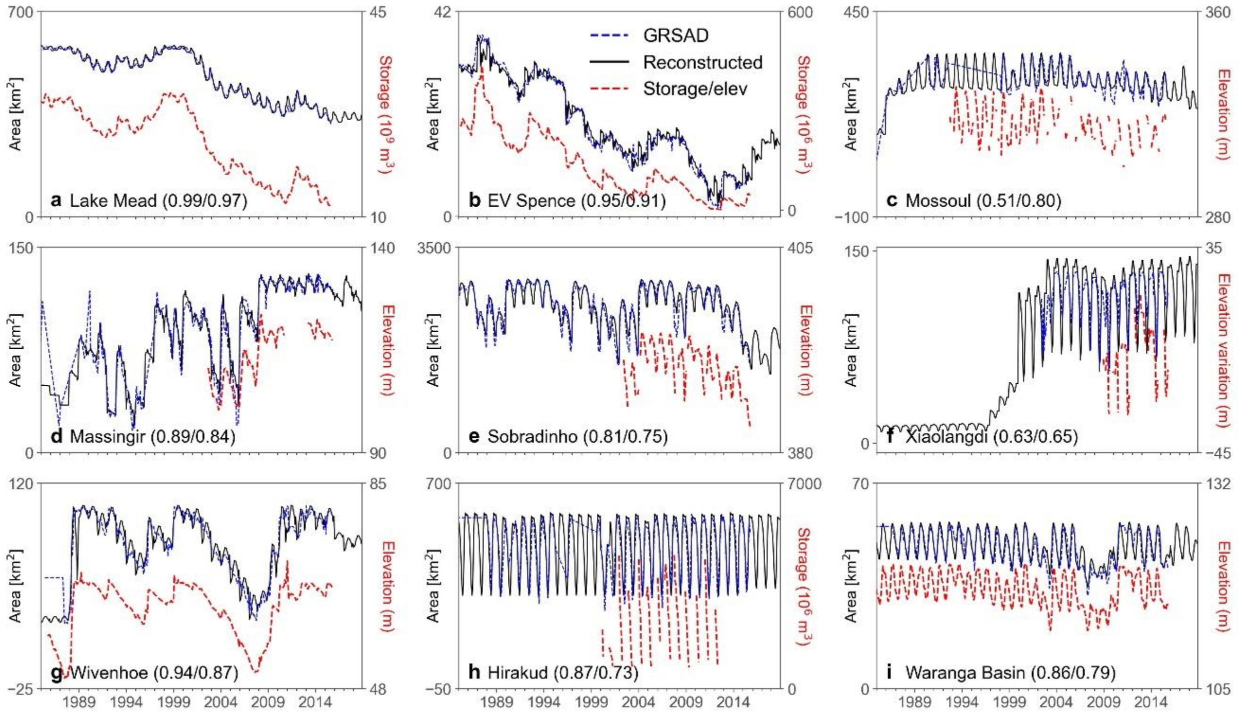

Supplementary Fig. 10.

Validation of reconstructed monthly area (this study) using observed reservoir storage or elevation time series for a) Lake Mead, b) EV Spence reservoir, c) Lake Mossoul, d) Massingir Lake, e) Sobradinho Reservoir, f) Xiaolangdi Reservoir, g) Lake Wivenhoe, h) Hirakud Reservoir, and i) Waranga Basin Reservoir. The time series from GRSAD (Zhao and Gao, 2018) is also plotted for reference. The two values in the parenthesis represent R^2 values for 1) GRSAD area and observed storage or elevation and 2) reconstructed area and observed storage or elevation.

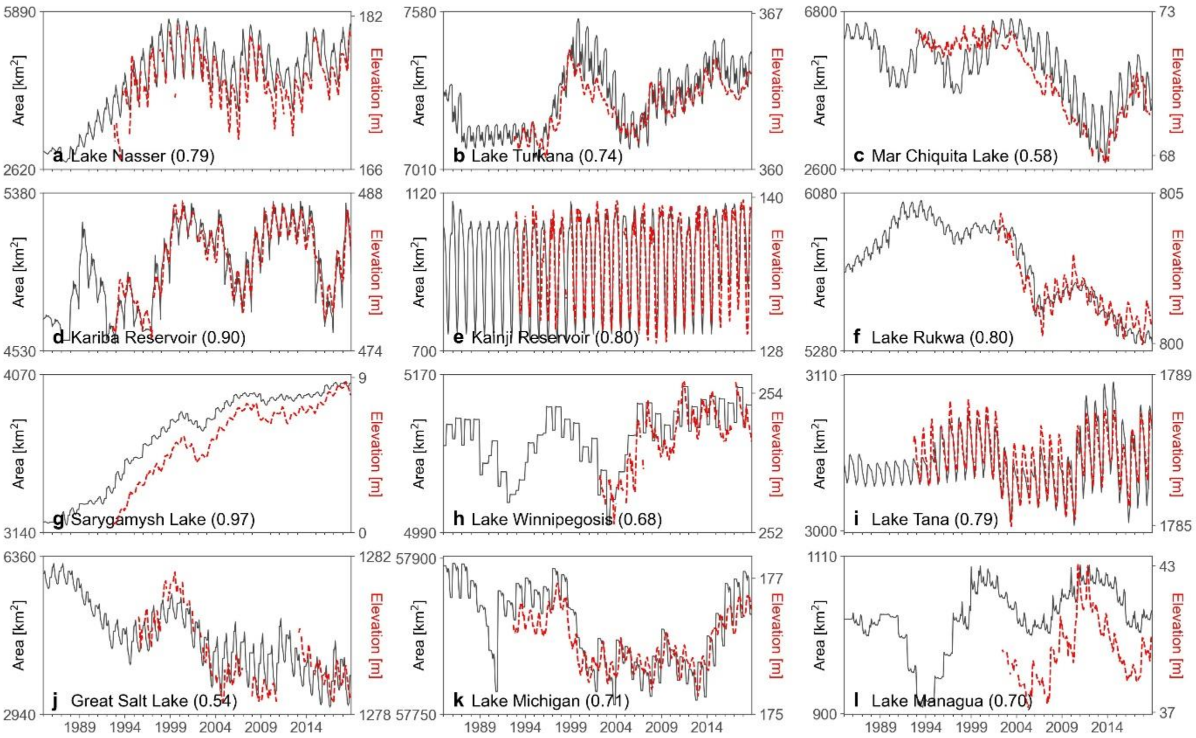

Supplementary Fig. 11.

Validation of reconstructed monthly area (this study) using satellite altimetry elevation time series for 12 large (> 1000 km^2) lakes including a) Lake Nasser, b) Lake Turkana, c) Mar Chiquita Lake, d) Kariba Reservoir, e) Kainji Reservoir, f) Lake Rukwa, g) Sarygamysh Lake, h) Lake Winnipegosis, i) Lake Tana, j) Great Salt Lake, k) Lake Michigan, and l) Lake Managua. The value in the parenthesis represents R^2 values for reconstructed area and altimetry elevation.

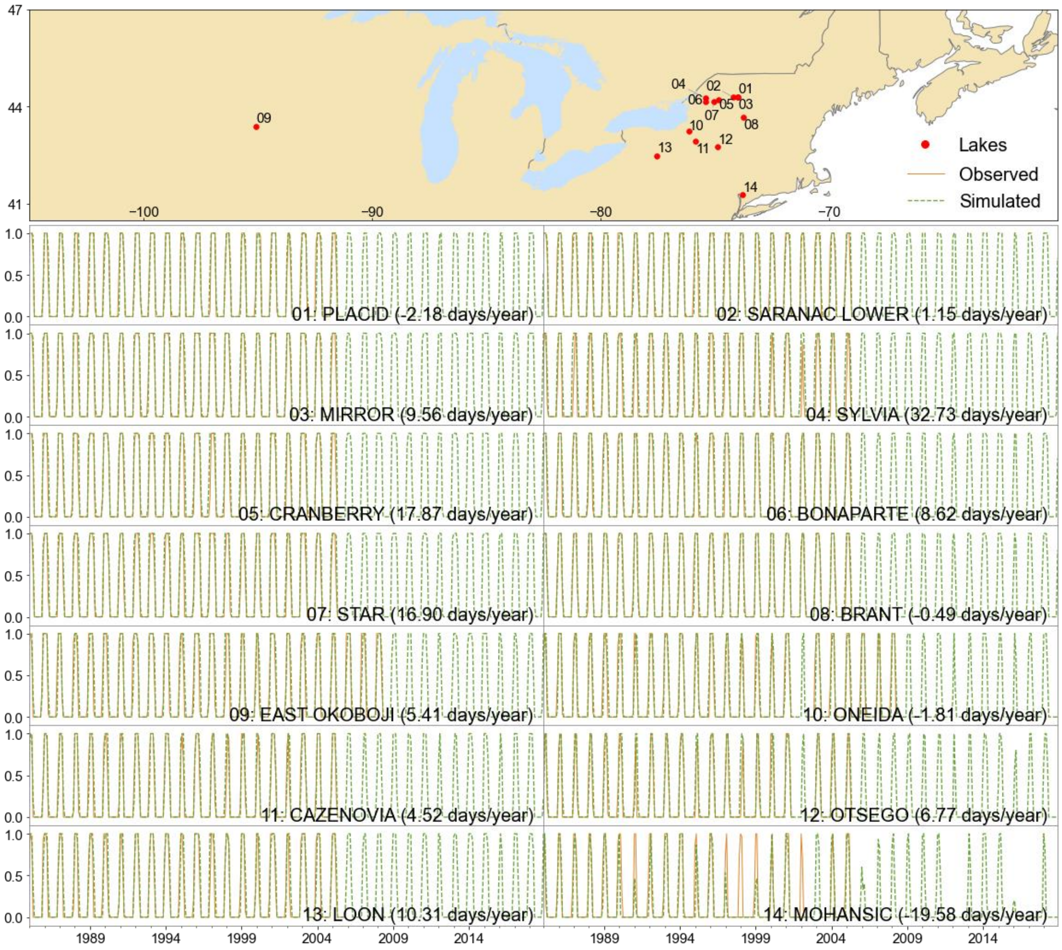

Supplementary Fig. 12.

Validation of monthly fraction of ice duration using long-term in-situ ice phenology data for 14 North American lakes. The in-situ data for ice duration were based on Benson et al. (2012).

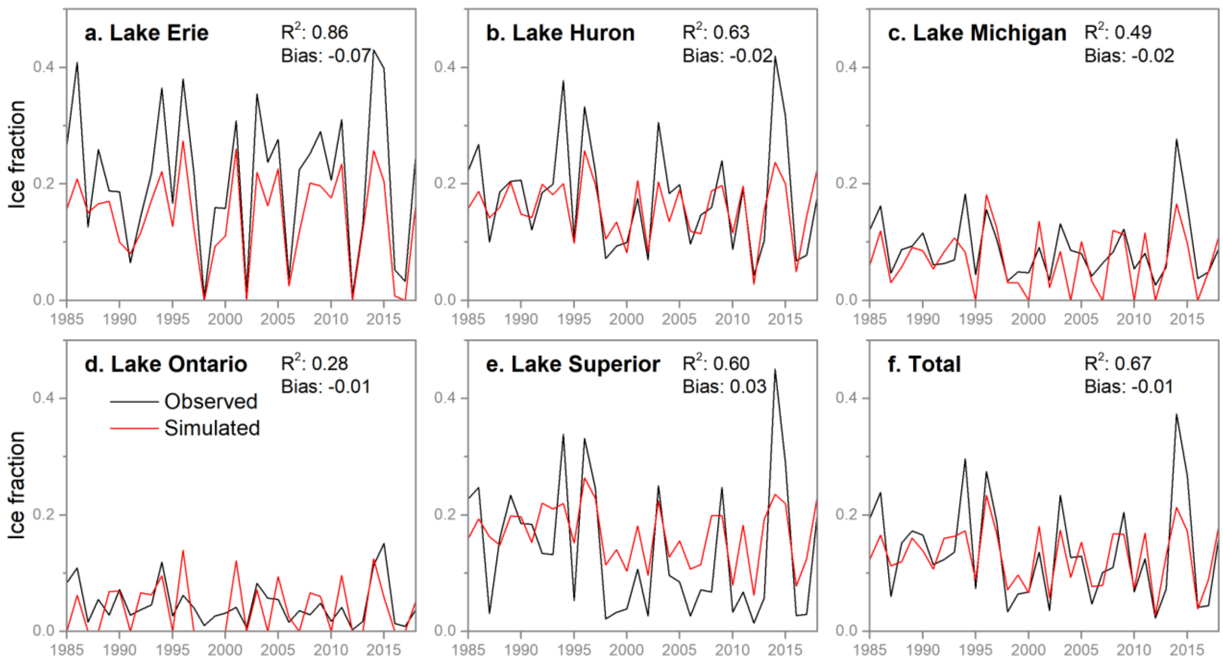

Supplementary Fig. 13.
Annual average ice fraction from the North American Great Lakes including a) Lake Erie, b) Lake Huron, c) Lake Michigan, d) Lake Ontario, e) Lake Superior, and f) the total of the five lakes. Observed values were collected from the Great Lakes Environmental Research Laboratory (https://www.glerl.noaa.gov/data/ice/).

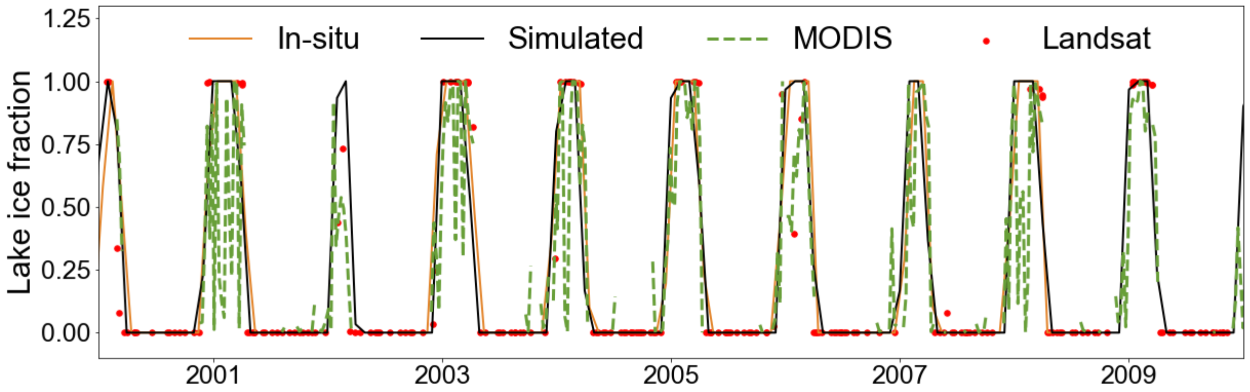

Supplementary Fig. 14.
Comparison of in-situ observed, MODIS (MOD10A2), Landsat (CFMask), and simulated lake ice phenology for Lake Oneida, USA. The in-situ data for ice duration were based on Benson et al. (2012).

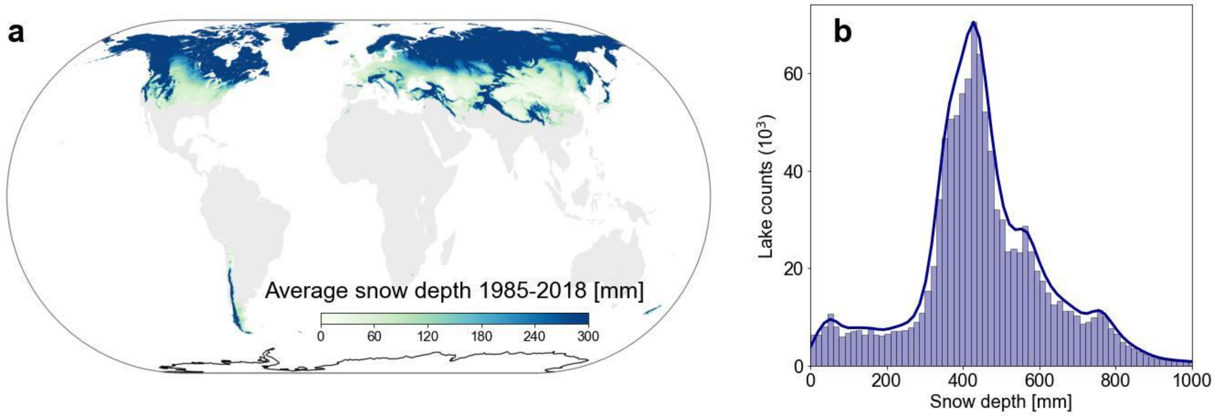

Supplementary Fig. 15.
Average snow coverage on a global scale. a) Average snow depth from 1985 to 2018 for months that have an average below-zero air temperature from the ERA5 dataset; b) Kernel density of snow depths on the lake surface for the 1.42 million lakes.

Supplementary Note 1

Lake ice sublimation

While the major focus of our study is the evaporation process, which by definition is “a type of vaporization that occurs on the surface of a liquid as it changes into the gas phase”, we would like to emphasize that lake ice sublimation can also contribute to the water losses for high-latitude and high-altitude lakes, especially in arid regions such as the Tibet Plateau. However, on a global scale, the lake ice sublimation can be much smaller than water evaporation due to snow coverage on lake ice and the relatively small sublimation rate.

In high-latitude and high-altitude regions, lake ice is mostly covered by snow in the winter season (Supplementary Fig. 15a). Such a snow layer can insulate the underneath ice and limit the direct heat transfer in the air-ice interface (Sturm and Liston, 2003). The accumulation of snow pressure in the snow-ice interface may also accommodate the formation of snow-ice and superimposed ice (Cheng et al., 2003; Semmler et al., 2012). These processes limit the lake ice sublimation rate. Meanwhile, ice sublimation can also be compensated by water vapor condensation when the atmospheric vapor pressure is larger than saturation pressure at ice temperature (Box and Steffen, 2001).

Direct measurements of lake ice sublimation are complicated by (1) snow cover and snow sublimation, (2) atmospheric vapor condensation, and (3) ice ablation (Sturm and Liston, 2003; Box and Steffen, 2001). Thus, it is difficult to get reliable sublimation rate measurements for lake ice. Based on glacier snowpack measurements, several studies report an average snow sublimation rate of 0.47 mm d^{-1} (Reba et al., 2012; Sexstone et al., 2016; Stössel et al., 2010; Stigter et al., 2018; Wagnon et al., 2003). Because lake ice has a much smaller specific surface area (SSA) than snow crystals (Matzl and Schneebeli, 2006), we would expect that lake ice sublimation rate is much smaller, leading to little impact on lake water losses.

Supplementary Table 1.

Long-term (1985-2018) average and trend for evaporation rate ($\boldsymbol{E_{lake}}$), open water area ($\boldsymbol{A_o}$), and evaporation volume ($\boldsymbol{V_E}$)

	$\boldsymbol{E_{lake}}$ (mm year^{-1})	$\boldsymbol{E_{lake}}$ trend (mm year^{-1} year^{-1})	$\boldsymbol{A_o}$ (km^2)	$\boldsymbol{A_o}$ trend (km^2 year^{-1})	$\boldsymbol{V_E}$ (km^3)	$\boldsymbol{V_E}$ trend (km^3 year^{-1})
**NF**	241	0.88	196914	758	134	0.60
**NC**	466	0.73	349940	693	266	0.48
**NT**	728	1.66	366311	104	293	-0.16
**NW**	1070	2.14	62462	115	68	0.20
**NH**	1353	2.16	65811	63	92	0.26
**TH**	1632	1.43	141661	478	228	0.94
**SH**	1636	1.55	212864	284	339	0.87
**SW**	1477	2.08	58000	-102	71	-0.08
**ST**	629	1.54	15601	7	10	0.02
**Global**	1077	1.57	1469564	2400	1500	3.12

Supplementary Table 2.
Buffering distance for HydroLAKES dataset.

Lake area (km^2)	Number of lakes	Buffer distance (m)
**$A \leq 0.1$***	56407	150
**$0.1 \leq A \leq 1$**	1186952	250
**$1 \leq A \leq 10$**	167645	500
**$10 \leq A \leq 100$**	14976	750
**$100 \leq A \leq 1000$**	1530	1000
**$A \geq 1000$**	177	1500

* Although the HydroLAKES dataset officially only reports lakes that are larger than 0.1 km^2, some reported polygons have areas that are smaller than 0.1 km^2 due to uncertainties from HydroLAKES data sources.

Supplementary Table 3.
Comparison of simulated evaporation with other studies.

Lake	Area (km^2)	Simulated evaporation ($mm\ d^{-1}$)	Evaporation from other sources ($mm\ d^{-1}$)	Reference
Lake Superior	82,100	1.33	1.37 (1.27 to 1.77)	Schertzer and Rao, 2009; Blanken et al., 2011
Lake Victoria	68,800	4.33	4.25	Yin et al., 2000
Aral Sea	68,000	2.90	2.18 to 3.30	Small et al., 1999
Lake Chad	18,750	6.00	5.95	Bouchez et al., 2016
Lake Nasser	5,250	5.95	5.95	Elsawwaf et al., 2010
Lake Okeechobee	1,900	4.00	3.95 to 4.38	Abtew, 2001

References

1. Abtew, W. (2001). Evaporation estimation for Lake Okeechobee in south Florida. Journal of Irrigation and Drainage Engineering, 127(3), 140-147.
2. Benson, B. J., Magnuson, J. J., Jensen, O. P., Card, V. M., Hodgkins, G., Korhonen, J., ... & Granin, N. G. (2012). Extreme events, trends, and variability in Northern Hemisphere lake-ice phenology (1855–2005). *Climatic Change*, *112*(2), 299-323.
3. Blanken, P. D., Spence, C., Hedstrom, N., & Lenters, J. D. (2011). Evaporation from Lake Superior: 1. Physical controls and processes. *Journal of Great Lakes Research*, *37*(4), 707-716.
4. Bouchez, C., Goncalves, J., Deschamps, P., Vallet-Coulomb, C., Hamelin, B., Doumnang, J. C., & Sylvestre, F. (2016). Hydrological, chemical, and isotopic budgets of Lake Chad: a quantitative assessment of evaporation, transpiration and infiltration fluxes. *Hydrology and Earth System Sciences*, *20*(4), 1599-1619.
5. Box, J. E., & Steffen, K. (2001). Sublimation on the Greenland ice sheet from automated weather station observations. *Journal of Geophysical Research: Atmospheres*, 106(D24), 33965-33981.
6. Cheng, B., Vihma, T., & Launiainen, J. (2003). Modelling of superimposed ice formation and subsurface melting in the Baltic Sea. *Geophysica*, *39*(1-2), 31-50.
7. Dugan, H. A., Obryk, M. K., & Doran, P. T. (2013). Lake ice ablation rates from permanently ice-covered Antarctic lakes. *Journal of Glaciology*, *59*(215), 491-498.
8. Elsawwaf, M., Willems, P., Pagano, A., & Berlamont, J. (2010). Evaporation estimates from Nasser Lake, Egypt, based on three floating station data and Bowen ratio energy budget. Theoretical and Applied Climatology, 100(3), 439-465.
9. Matzl, M., & Schneebeli, M. (2006). Measuring specific surface area of snow by near-infrared photography. *Journal of Glaciology*, 52(179), 558-564.
10. Moreo MT. Evaporation Data from Lake Mead and Lake Mohave, Nevada and Arizona, March 2010 through April 2015: U.S. Geological Survey Data Release, http://dx.doi.org/10.5066/F79C6VG3.). US Geological Survey (2015).
11. Wagnon, P., Sicart, J. E., Berthier, E., & Chazarin, J. P. (2003). Wintertime high-altitude surface energy balance of a Bolivian glacier, Illimani, 6340 m above sea level. *Journal of Geophysical Research: Atmospheres*, 108(D6).
12. Reba, M. L., Pomeroy, J., Marks, D., & Link, T. E. (2012). Estimating surface sublimation losses from snowpacks in a mountain catchment using eddy covariance and turbulent transfer calculations. *Hydrological Processes*, *26*(24), 3699-3711.
13. Small, E. E., Sloan, L. C., Hostetler, S., & Giorgi, F. (1999). Simulating the water balance of the Aral Sea with a coupled regional climate-lake model. *Journal of Geophysical Research: Atmospheres*, *104*(D6), 6583-6602.
14. Schertzer, W. M., & Rao, Y. R. (2009). An overview of the characteristics of Lake Superior meteorology, hydrology and physical limnology. *State of Lake Superior*, 38.
15. Sturm, M., & Liston, G. E. (2003). The snow cover on lakes of the Arctic Coastal Plain of Alaska, USA. *Journal of Glaciology*, *49*(166), 370-380.
16. Semmler, T., Cheng, B., Yang, Y., & Rontu, L. (2012). Snow and ice on Bear Lake (Alaska)–sensitivity experiments with two lake ice models. *Tellus A: Dynamic Meteorology and Oceanography*, *64*(1), 17339.
17. Stössel, F., Guala, M., Fierz, C., Manes, C., & Lehning, M. (2010). Micrometeorological and morphological observations of surface hoar dynamics on a mountain snow cover. *Water Resources Research*, *46*(4).

18. Sexstone, G. A., Clow, D. W., Stannard, D. I., & Fassnacht, S. R. (2016). Comparison of methods for quantifying surface sublimation over seasonally snow-covered terrain. *Hydrological Processes*, *30*(19), 3373-3389.
19. Stigter, E. E., Litt, M., Steiner, J. F., Bonekamp, P. N., Shea, J. M., Bierkens, M. F., & Immerzeel, W. W. (2018). The importance of snow sublimation on a Himalayan glacier. *Frontiers in Earth Science*, *6*, 108.
20. Trabucco, A.; Zomer, R. (2019). Global Aridity Index and Potential Evapotranspiration (ET0) Climate Database v2. figshare. Fileset. https://doi.org/10.6084/m9.figshare.7504448.v3
21. Wild, M., Ohmura, A., Schär, C., Müller, G., Folini, D., Schwarz, M., ... & Sanchez-Lorenzo, A. (2017). The Global Energy Balance Archive (GEBA) version 2017: A database for worldwide measured surface energy fluxes. *Earth System Science Data*, *9*(2), 601-613.
22. Yin, X., Nicholson, S. E., & Ba, M. B. (2000). On the diurnal cycle of cloudiness over Lake Victoria and its influence on evaporation from the lake. *Hydrological Sciences Journal*, *45*(3), 407-424.
23. Zhang, Z., Zhang, M., Cao, C., Wang, W., Xiao, W., Xie, C., ... & Lee, X. (2020). A dataset of microclimate and radiation and energy fluxes from the Lake Taihu eddy flux network. *Earth System Science Data*, *12*(4), 2635-2645.
24. Zhao, G., & Gao, H. (2018). Automatic correction of contaminated images for assessment of reservoir surface area dynamics. *Geophysical Research Letters*, *45*(12), 6092-6099.
